# Polymorphism of rs873308 near the transmembrane protein 57 gene is associated with serum lipid levels

**DOI:** 10.1042/BSR20130131

**Published:** 2014-03-14

**Authors:** Tao Guo, Rui-Xing Yin, Quan-Zhen Lin, Jian Wu, Shao-Wen Shen, Jia-Qi Sun, Guang-Yuan Shi, Jin-Zhen Wu, Hui Li, Yi-Ming Wang

**Affiliations:** *Department of Cardiology, Institute of Cardiovascular Diseases, the First Affiliated Hospital, Guangxi Medical University, 22 Shuangyong Road, Nanning 530021, Guangxi, People's Republic of China; †Clinical Laboratory of the Affiliated Cancer Hospital, Guangxi Medical University, Nanning 530021, Guangxi, People's Republic of China; ‡College of Stomatology, Guangxi Medical University, 22 Shuangyong Road, Nanning 530021, Guangxi, People's Republic of China

**Keywords:** environmental factors, lipids, single-nucleotide polymorphism, transmembrane protein 57, Apo, apolipoprotein, BMI, body mass index, BP, blood pressure, CHD, coronary heart disease, GWAS, genome-wide association study, HDL-C, high-density lipoprotein cholesterol, LDL, low-density lipoprotein, LDL-C, low-density lipoprotein cholesterol, RFLP, restriction-fragment-length polymorphism, SNP, single-nucleotide polymorphism, TC, total cholesterol, TAG, triacylglycerol, TMEM, transmembrane protein

## Abstract

SNP (single-nucleotide polymorphism) of rs10903129 near the TMEM (transmembrane protein) 57 locus has been associated with TC (total cholesterol) in a previous GWAS (genome-wide association study), but the association of TMEM57 rs873308 SNP and serum lipid levels has not been previously reported. The current study was undertaken to detect the association of the TMEM57 rs873308 SNP and several environmental factors with serum lipid profiles in the Han Chinese and Mulao populations. The genotypes of the TMEM57 rs873308 SNP in 865 individuals of Han Chinese and 902 participants of Mulao nationality were determined by PCR and RFLP (restriction-fragment-length polymorphism) combined with gel electrophoresis and then confirmed by direct sequencing. The T allele frequency of TMEM57 rs873308 SNP was not different between Han and Mulao (23.18% versus 25.72%, *P*>0.05), but different between males and females in the two ethnic groups (*P*<0.05). The T allele carriers had lower serum TC, Apo (apolipoprotein) B, HDL-C (high-density lipoprotein cholesterol) levels, ApoA1/ApoB ratio in Han; and lower TAG (triacylglycerol), LDL-C (low-density lipoprotein cholesterol), ApoA1 levels and the ApoA1/ApoB ratio and higher HDL-C levels in Mulao than the T allele non-carriers. There was also different association of the TMEM57 rs873308 SNP and serum lipid profiles between males and females in the both ethnic groups. Serum lipid parameters in the two ethnic groups were also associated with several environmental factors. The association of the TMEM57 rs873308 SNP and serum lipid levels was different in the Han Chinese and Mulao populations and between males and females in the both ethnic groups. There may be a sex-specific association of the TMEM57 rs873308 SNP and serum lipid levels in our study populations.

## INTRODUCTION

CHD (coronary heart disease) is one of the world's top ‘economic killer’ as well as its likely leading cause of death in the world [1]. The estimated direct cost of CHD in 2010 was $272.5 billion, and it is projected to reach $818 billion by 2030 in America [2]. It is a universally acknowledged truth that dyslipidaemia is presumed to play a vital role in someone susceptible to CHD [3]. Serum or plasma TC (total cholesterol) [4], TAG (triacylglycerol) [5], HDL-C (high-density lipoprotein cholesterol) [6], LDL-C (low-density lipoprotein cholesterol) [7], Apo (apolipoprotein) A1 [8], ApoB [9] and ApoA1/ApoB ratio [10] were traditionally monitored as predictors of CHD events and the main target for therapeutic intervention.

Recent research has indicated that dyslipidaemia is influenced by genetic factors [11]. Most genetic effects are modest in size, and the vast majority of the heritability of dyslipidaemia remains unexplained. Many of the known dyslipidaemia susceptible loci come from GWASs (genome-wide association studies) of common SNPs (single-nucleotide polymorphisms). Another promising approach to identify the dyslipidaemia biomarkers is from studies of the gene expression signatures of dyslipidaemia, which have yielded promising results, but to date only a few genes are common across studies. More thorough gene expression studies of dyslipidaemia are needed for new discoveries and to corroborate previous findings. In addition, dyslipidaemia is well recognized as a complex trait caused by multiple environmental factors and the interaction of genetic and environmental factors.

TMEM (transmembrane protein) 57, a variant in TMEM family member, plays a major role in the trafficking and lipid metabolism. It is located on human chromosome 1. There are four adjacent SNPs of rs873308, rs11802413, rs7541095 and rs10903129 in the TMEM57 (http://hapmap.ncbi.nlm.nih.gov/). After screening the genome for common variants associated with serum lipids in ≥1000 000 individuals of European ancestry, Aulchenko *et al.* [12] studied TC associated genetic markers and identified the loci significantly associated with the trail: the corresponded to TMEM57. The gene is also implicated in other lipid traits. GWASs have identified genetic variant of the 10903129 SNP near TMEM57 loci associated with serum TC level [12,13]. However, the effect of rs873308 SNP near TMEM57 loci on serum lipid levels is not functionally validated and the mechanism is yet unclear. Furthermore, the reproducibility of this association has not been detected in the Chinese populations so far.

Since ancient times China is a multi-ethnic country. Among 56 ethnic groups in China, Han is the largest one, and Mulao (also known as Mulam) is one of the 55 minorities with a population of 207,352 according to the fifth national census statistics of China in 2000. Ninety percent of them live in the Luocheng Mulao Autonomous County, Guangxi Zhuang Autonomous Region. The history of this minority can be traced back to the Jin Dynasty (AD 265–420). One previous study had shown that the genetic relationship between Mulao nationality and other minorities in Guangxi was much closer than that between Mulao and Han or Uighur nationality [14]. In several previous studies, we have shown significant association of several SNPs [15–17] and serum lipid levels in the Mulao population. To the best of our knowledge, the association of rs873308 SNP and serum lipid levels has not been previously explored in the Chinese populations. Therefore, the aim of the present study was to assess the association of TMEM57 rs873308 SNP and several environmental factors with serum lipid phenotypes in the Han and Mulao populations.

## MATERIALS AND METHODS

### Study population

A total of 902 subjects of Mulao (446 males, 49.45% and 456 females, 50.55%) and 865 participants of Han Chinese (423 men, 48.90% and 442 women, 51.10%) were randomly selected from our previous stratified randomized samples. They reside in Luocheng Mulao Autonomous County, Guangxi Zhuang Autonomous Region, People's Republic of China. The age of the subjects ranged from 15 to 80 years. The mean age of Mulao participants was 52.48±14.70 years, whereas that of Han subjects was 52.40±14.04 years. All participants were essentially healthy rural agricultural workers, and had no evidence of diseases like atherosclerosis, CHD and diabetes. Any participant who had a history of taking medications known to affect serum lipid levels (lipid-lowering drugs such as statins or fibrates, β-blockers, diuretics, or hormones) was excluded before the blood sample was taken. The study design was approved by the Ethics Committee of the First Affiliated Hospital, Guangxi Medical University. Informed consent was obtained from all participants.

### Epidemiological survey

The survey was carried out using internationally standardized methods, following a common protocol [18]. Information on demographics, socio-economic status and lifestyle factors was collected with standardized questionnaires. Alcohol consumption was quantified as the number of liangs (about 50 g) of rice wine, corn wine, rum, beer or liquor consumed during the preceding 12 months. Alcohol consumption was categorized into groups of grams of alcohol per day: 0 (non-drinker),<25 and ≥ 25. Cigarette smoking status was categorized into groups of cigarettes per day: 0 (non-drinker),<20 and ≥ 20. In the physical examination, several parameters covering body height, weight, and waist circumference were measured. Sitting BP (blood pressure) was measured three times with the use of a mercury sphygmomanometer after about 5 min rest, and the average of the three measurements was used. Systolic BP was determined by the first Korotkoff sound, and diastolic BP by the fifth Korotkoff sound. Body weight, to the nearest 50 g, was estimated by a portable weighing machine. Height was measured, to the nearest 0.5 cm, using a stadiometer. From these two measurements BMI (body mass index, kg/m^2^) was calculated. Waist circumference was measured by a non-stretchable measuring tape.

### Biochemical measurements

Venous blood samples of 5 mL were drawn after at least 12 h of fasting. Two-fifths of the sample (2 mL) was collected in glass tubes and used to determine serum lipid levels. The remaining three-fifths of the sample (3 mL) was transferred to tubes with anticoagulants (4.80 g/l citric acid, 14.70 g/l glucose and 13.20 g/l trisodium citrate) and used to extract DNA. Measurements of serum TC, TAG, HDL-C, and LDL-C levels in the samples were performed by enzymatic methods with commercially available kits (RANDOX Laboratories Ltd., BT29 4QY; Daiichi Pure Chemicals Co., Ltd.). Serum ApoA1 and ApoB levels were detected by the immunoturbidimetric immunoassay using a commercial kit (RANDOX Laboratories Ltd.). All determinations were performed with an auto-analyser (Type 7170A; Hitachi Ltd.) in the Clinical Science Experiment Center of the First Affiliated Hospital, Guangxi Medical University [15–17].

### DNA amplification and genotyping

Genomic DNA of the samples was isolated from peripheral blood leucocytes according to the phenol–chloroform method [15–17]. The extracted DNA was stored at 4°C until analysis. Genotyping of the TMEM57 rs873308 SNP was determined by PCR and RFLP (restriction-fragment-length polymorphism). PCR amplification was performed using 5′- ACAAGAGCATGTGCAAGGTG -3′ and 5′-CCGGTAGAGAAAGACAACAGG-3′ (Sangon) as the forward and reverse primer pairs, respectively. Each reaction system of total volume of 25 ml, containing 10×PCR buffer (1.8 mM MgCl_2_) 2.5 μl, 1 U *Taq* polymerase, 2.5 mmol/l of each d-NTP (Tiangen) 2.0 μl, 20 pmol/l of each primer and 50 ng of genomic DNA; processing started at 95°C for 7 min and followed by 45 s of denaturing at 95°C, 45 s of annealing at 65°C and 1 min of elongation at 72°C for 33 cycles. The amplification was completed by final extension at 72°C for 7 min. After electrophoresis on a 2.0% (w/v) agarose gel with 0.5 μg/ml ethidium bromide, the amplified products were visualized under UV light. Then each restriction enzyme reaction was performed with 5 μl of amplified DNA; 7.5 μl of nuclease-free water and 1 μl of 10×buffer solution; and 5 units of HindIII restriction enzyme in a total volume of 15 μl digested at 37°C overnight. After restriction enzyme digestion of the amplified DNA, genotypes were identified by electrophoresis on 2% (w/v) ethidium-bromide-stained agarose gels and visualizing with UV illumination. Genotypes were scored by an experienced reader blinded to the epidemiological and serum lipid results.

### DNA sequencing

Six samples (CC, CT and TT genotypes in two, respectively) detected by the PCR–RFLP were also confirmed by direct sequencing. The PCR product was purified by low melting point gel electrophoresis and phenol extraction, and then the DNA sequences were analysed in Shanghai Sangon Biological Engineering Technology & Services Co., Ltd.

### Diagnostic criteria

The normal values of serum TC, TAG, HDL-C, LDL-C, ApoA1, ApoB levels and the ApoA1/ApoB ratio in our Clinical Science Experiment Center were 3.10–5.17, 0.56–1.70, 1.16–1.42, 2.70–3.10 mmol/l, 1.20–1.60, 0.80–1.05 g/l and 1.00–2.50, respectively. The individuals with TC>5.17 mmol/l and/or TAG>1.70 mmol/l were defined as hyperlipidaemic [19,20]. Hypertension was diagnosed according to the 1999 and 2003 criteria of the World Health Organization–International Society of Hypertension Guidelines for the management of hypertension [21–23]. The diagnostic criteria of overweight and obesity were according to the Cooperative Meta-analysis Group of China Obesity Task Force. Normal weight, overweight and obesity were defined as a BMI<24, 24–28 and>28 kg/m^2^, respectively [24].

### Statistical analyses

Epidemiological data were recorded on a pre-designed form and managed with Excel software. All calculations were performed using the software SPSS version 19.0 (SPSS Inc.) statistical package. Means±S.D. (serum TAG levels were presented as medians and interquartile ranges) and frequencies of baseline characteristics were calculated. Comparison of numerical variables, such as age and BMI, between the both groups was tested by the Student's unpaired *t* test. Categorical variables were analysed with χ^2^ tests or Fisher's exact test. Allele frequency was determined via direct counting, and the standard goodness-of-fit test was used to test the Hardy–Weinberg equilibrium. Difference in genotype distribution between the groups was determined by the χ^2^ test. The association of genotypes and serum lipid parameters was determined using ANCOVA (analysis of co-variance). Sex, age, BMI, BP, alcohol consumption, cigarette smoking were adjusted for the statistical analysis. Multivariate linear regression analysis with stepwise modeling was performed to evaluate the association of serum lipid levels with genotypes (CC=1, CT=2 and TT=3) and several environmental factors in the combined population of Mulao and Han, Mulao, Han, males and females, respectively. A *P* value of <0.05 was considered statistically significant.

## RESULTS

### General characteristics and serum lipid levels

Comparison of the general characteristics and serum lipid levels between the Han and Mulao populations is summarized in Table 1. Body weight, BMI, percentages of cigarette smoking and alcohol consumption, diastolic BP, blood glucose, serum TC, TAG and ApoA1 levels were higher in Han than in Mulao (*P*<0.05–0.001), whereas the levels of HDL-C and ApoB was lower in Han than in Mulao (*P*<0.05–0.001). There were no significant differences in the gender ratio, age structure, body height, waist circumference, systolic BP, pulse pressure, serum LDL-C levels and the ApoA1/ApoB ratio between the two ethnic groups (*P*>0.05 for all).

**Table 1:** Comparison of demographic, lifestyle characteristics and serum lipid levels between the Han and Mulao populations HDL-C, high-density lipoprotein cholesterol; LDL-C, low-density lipoprotein cholesterol. The value of TAG was presented as median (interquartile range). The difference between the two ethnic groups was determined by the Wilcoxon-Mann-Whitney test.

Parameter	Han	Mulao	*t* (***χ*^2^**)	*P*
Number	865	902		
Male/female	423/442	446/456	0.229	0.819
Age (years)	52.40±14.04	52.48±14.70	−0.117	0.907
Height (cm)	155.09±7.95	155.12±7.93	−0.086	0.932
Weight (kg)	54.27±9.21	52.62±9.45	3.712	0.000
BMI (kg/m^2^)	22.53±3.33	21.80±3.11	4.768	0.000
Waist circumference (cm)	75.86±7.96	75.13±8.54	1.876	0.061
Cigarette smoking [*n* (%)]				
Non-smoker	591(68.32)	693(76.83)		
<20 cigarettes/day	106(12.26)	74(8.20)	3.557	0.000
≥20 cigarettes/day	168(19.42)	135(14.97)		
Alcohol consumption [*n* (%)]				
Non-drinker	648(74.91)	695(77.05)		
<25 g/day	61(7.05)	89(9.87)	2.012	0.044
≥25 g/day	156(18.04)	118(13.08)		
Systolic BP (mmHg)	130.87±18.57	129.56±22. 07	1.348	0.178
Diastolic BP (mmHg)	82.80±10.81	80.99±11.53	3.400	0.001
Pulse pressure (mmHg)	48.08±14.18	48.58±17.05	−0.668	0.504
Glucose (mmol/l)	6.23±1.97	6.03±1.68	2.240	0.025
Total cholesterol (mmol/l)	5.15±1.23	5.01±1.28	2.276	0.023
TAG (mmol/l)	1.13(0.82)	1.04(0.75)	−8.674	0.000
HDL-C (mmol/l)	1.73±0.57	1.78±0.50	−2.015	0.044
LDL-C (mmol/l)	2.89±0.92	2.91±0.92	−0.377	0.706
ApoA1 (g/l)	1.36±0.27	1.30±0.45	3.110	0.002
ApoB (g/l)	0.88±0.22	1.02±0.63	−6.134	0.000
ApoA1/ApoB	1.63±0.51	1.59±1.16	0.858	0.391

### Results of electrophoresis and genotyping

**Figure 1:**
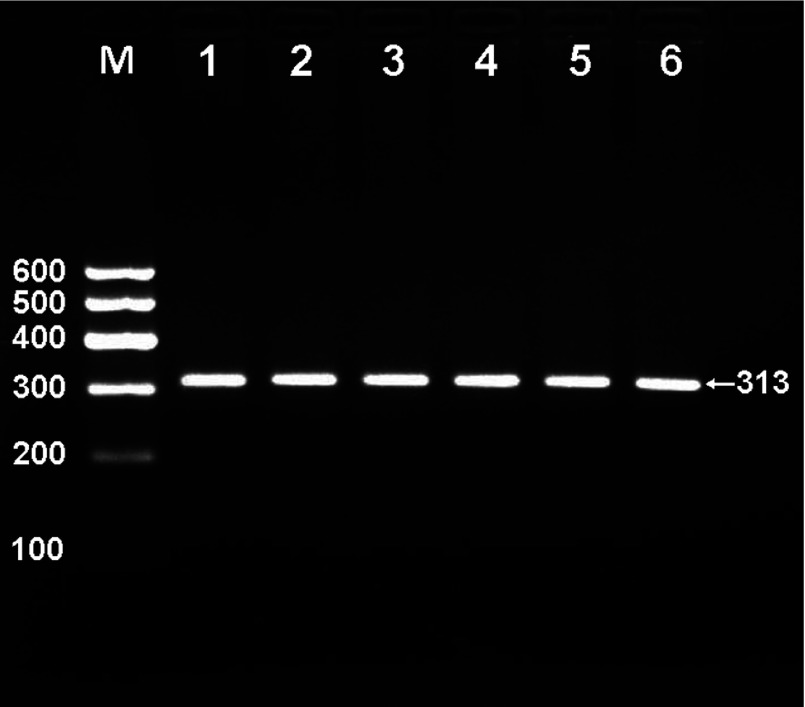
Electrophoresis of PCR products of the samples Lane M is the 100 bp Marker ladder; lanes 1–6 are the samples, the 313 bp bands are the target genes.

**Figure 2:**
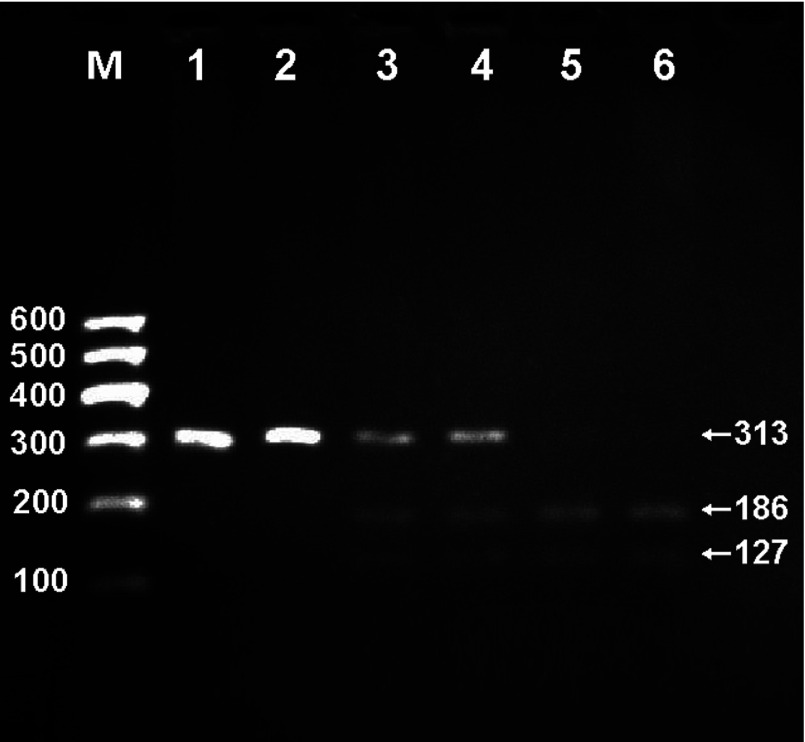
Genotyping of the TMEM57 rs873308 SNP Lane M is the 100 bp Marker ladder; lanes 1 and 2, CC genotype (313-bp); lanes 3 and 4, CT genotype (313-, 186- and 127-bp); and lanes 5 and 6, TT genotype (186- and 127-bp).

After the genomic DNA of the samples was amplified by PCR and imaged by 2% agarose gel electrophoresis, the products of 313-bp nucleotide sequences were found in all samples (Figure 1). The genotypes identified were named according to the presence (T allele) or absence (C allele) of the enzyme restriction sites. Thus, TT genotype was homozygote for the presence of the site (bands at 186- and 127-bp), CT genotype was heterozygote for the presence and absence of the site (bands at 313-, 186- and 127-bp), and CC genotype was homozygote for the absence of the site (bands at 313-bp; Figure 2). The genotypes of the rs873308 SNP were followed by the Hardy–Weinberg equilibrium.

### Results of sequencing

**Figure 3:**
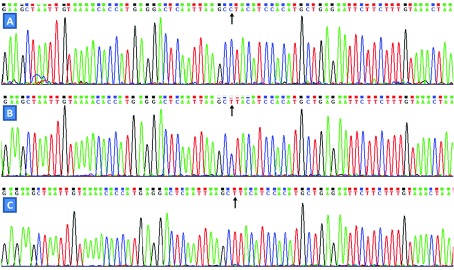
A part of the nucleotide forward sequence of the TMEM57 rs873308 SNP (A) CC genotype; (B) CT genotype; (C) TT genotype.

The results were shown as CC, CT and TT genotypes by PCR–RFLP, the CC, CT and TT genotypes were also confirmed by direct sequencing (Figure 3), respectively.

### Genotypic and allelic frequencies

The genotypic and allelic frequencies of rs873308 SNP near the TMEM57 are shown in Table 2. The frequencies of C and T alleles were 76.82 and 23.18% in Han, and 74.28 and 25.72% in Mulao (*P*>0.05), respectively. The frequency of T allele in males and females was 21.04% versus 25.23% in Han, and 23.01% versus 28.29% in Mulao (*P*<0.05 for each), respectively. There were no differences in the genotypic frequencies of TMEM57 rs873308 SNP between Han and Mulao or between males and females in the two ethnic groups.

**Table 2:** Comparison of the genotype and allele frequencies of TMEM57 rs873308 SNP in the Han and Mulao populations [*n* (%)]

Group	*n*	Genotype			Allele	
		CC	CT	TT	C	T
Han	865	566(65.43)	197(22.78)	102(11.79)	1329(76.82)	401(23.18)
Mulao	902	555(61.53)	230(25.50)	117(12.97)	1340(74.28)	464(25.72)
***χ*** ^2^			2. 912		3.086	
*P*			0.233		0.079	
Han						
Male	423	285(67.37)	98(23.17)	40(9.46)	668(78.96)	178(21.04)
Female	442	281(63.57)	99(22.40)	62(14.03)	661(74.77)	223(25.23)
***χ*** ^2^			4.363		4.254	
*P*			0.113		0.039	
Mulao						
Male	446	290(65.02)	106(23.77)	50(11.21)	686(76.91)	206(23.09)
Female	456	265(58.10)	124(27.19)	67(14.69)	654(71.71)	258(28.29)
***χ*** ^2^			4.895		6.371	
*P*			0.087		0.012	

### Genotypes and serum lipid levels

As shown in Tables 3 and 4, serum TC, HDL-C, ApoB levels and the ApoA1/ApoB ratio in Han were different among the genotypes (*P*<0.05–0.001), the T allele carriers had lower serum TC and ApoB levels and higher HDL-C levels and the ApoA1/ApoB ratio than the T allele non-carriers. Serum TAG, HDL-C, LDL-C, ApoA1 levels and the ApoA1/ApoB ratio were different among the genotypes in Mulao (*P*<0.05–0.001), the T allele carriers had lower serum TAG, LDL-C, ApoA1 levels and the ApoA1/ApoB ratio and higher serum HDL-C levels than the T allele non-carriers. Subgroup analyses showed that serum TC, TAG, HDL-C, ApoB levels and the ApoA1/ApoB ratio in Han males and serum TAG, HDL-C, ApoA1 levels and the ApoA1/ApoB ratio in Han females were different among the genotypes (*P*<0.05–0.001); the T allele carriers had lower serum TC, TAG and ApoB levels and higher serum HDL-C level and the ApoA1/ApoB ratio than the T allele non-carriers in Han males (*P*<0.05–0.001). Meanwhile, the T allele carriers had lower serum TAG levels and higher serum HDL-C, ApoA1 levels and the ApoA1/ApoB ratio than the T allele non-carriers in Han females (*P*<0.05–0.001). Accordingly, serum LDL-C and ApoA1 levels in Mulao males and serum TAG, HDL-C and ApoA1 levels in Mulao females were different among the genotypes (*P*<0.05–0.001). The T allele carriers had lower serum LDL-C and ApoA1 levels than the T allele non-carriers in Mulao males (*P*<0.05–0.001), and the T allele carriers had lower serum TAG and ApoA1 levels and higher serum HDL-C level than the T allele non-carriers in Mulao females (*P*<0.05–0.001).

**Table 3:** Comparison of the genotypes and serum lipid levels in the Han and Mulao populations TC, total cholesterol; TAG, triacylglycerol; HDL-C, high-density lipoprotein cholesterol; LDL-C, low-density lipoprotein cholesterol; ApoA1, apolipoprotein A1; ApoB, apolipoprotein B; ApoA1/ApoB, the ratio of apolipoprotein A1 to apolipoprotein B. The value of TAG was presented as median (interquartile range). The difference between the genotypes was determined by the Wilcoxon-Mann-Whitney test.

Ethnic Genotype	*N*	TC (mmol/l)	TAG (mmol/l)	HDL-C (mmol/l)	LDL-C (mmol/)	Apo A1 (g/l)	ApoB (g/l)	ApoA1/ApoB
Han								
CC	566	5.23±1.37	1.14(0.86)	1.68±0.43	2.92±1.00	1.35±0.27	0.89±0.23	1.59±0.49
CT	197	5.02±0.86	1.12(0.78)	1.73±0.41	2.91±0.76	1.36±0.26	0.87±0.19	1.63±0.51
TT	102	4.92±0.9	0.98(0.70)	2.00±1.14	2.72±0.70	1.40±0.27	0.82±0.21	1.82±0.60
*F*		4.073	−0.064	14.725	1.974	2.021	4.750	8.280
*P*		0.017	0.949	0.000	0.140	0.133	0.009	0.000
Mulao								
CC	555	5.06±1.22	2.88(2.39)	1.74±0.43	2.97±0.89	1.35±0.39	1.00±0.58	1.66±1.29
CT	230	4.95±1.16	2.86(2.31)	1.83±0.55	2.87±0.87	1.32±0.48	1.07±0.71	1.64±0.85
TT	117	4.88±1.69	2.68(2.15)	1.86±0.65	2.72±1.09	1.26±0.58	1.02±0.67	1.56±0.98
*F*		1.386	−6.446	4.582	3.810	7.813	1.127	3.028
*P*		0.251	0.000	0.010	0.023	0.000	0.324	0.049

**Table 4:** Comparison of the genotypes and serum lipid levels between males and females in the Han and Mulao populations TC, total cholesterol; TAG, triacylglycerol HDL-C, high-density lipoprotein cholesterol; LDL-C, low-density lipoprotein cholesterol; ApoA1, apolipoprotein A1; ApoB, apolipoprotein B; ApoA1/ApoB, the ratio of apolipoprotein A1 to apolipoprotein B. The value of TAG was presented as median (interquartile range). The difference between the genotypes was determined by the Wilcoxon-Mann-Whitney test.

Ethnic/Genotype	N	TC (mmol/l)	TAG (mmol/l)	HDL-C (mmol/l)	LDL-C (mmol/l)	ApoA1 (g/l)	ApoB (g/l)	ApoA1/ApoB
Han/male								
CC	285	5.47±1.41	1.32(0.94)	1.64±0.42	2.95±1.06	1.38±0.29	0.95±0.23	1.53±0.48
CT	98	5.07±0.82	1.18(0.78)	1.74±0.42	2.89±0.69	1.39±0.29	0.90±0.18	1.61±0.51
TT	40	5.01±0.83	1.00(0.79)	1.77±0.51	2.81±0.63	1.40±0.32	0.86±0.20	1.72±0.60
*F*		5.325	−3.539	3.055	0.451	0.202	4.475	3.291
*P*		0.005	0.000	0.048	0.638	0.817	0.012	0.038
Han/female								
CC	281	4.98±1.27	1.02(0.80)	1.71±0.43	2.89±0.95	1.31±0.25	0.83±0.21	1.66±0.49
CT	99	4.98±0.91	1.00(0.71)	1.72±0.40	2.92±0.82	1.33±0.23	0.86±0.19	1.64±0.51
TT	62	4.86±0.98	0.87(0.65)	2.16±1.39	2.67±0.75	1.40±0.25	0.79±0.21	1.88±0.60
*F*		0.289	−3.653	12.069	1.797	3.387	1.724	4.961
*P*		0.749	0.000	0.000	0.167	0.035	0.180	0.007
Mulao/male								
CC	290	5.11±1.24	1.13(0.83)	1.73±0.45	2.92±0.85	1.35±0.42	1.04±0.65	1.61±1.23
CT	106	4.82±1.23	1.02(0.69)	1.75±0.64	2.78±0.88	1.20±0.46	1.09±0.75	1.41±0.79
TT	50	4.98±1.76	1.05(0.67)	1.82±0.69	2.56±1.09	1.19±0.55	0.99±0.63	1.71±0.93
*F*		1.995	−1.106	0.660	3.899	5.216	0.357	1.698
*P*		0.137	0.269	0. 517	0.021	0.006	0.700	0.184
Mulao/female								
CC	265	5.01±1.21	1.01(0.76)	1.75±0.40	3.02±0.94	1.34±0.36	0.95±0.50	1.72±1.35
CT	124	5.06±1.09	0.96(0.75)	1.90±0.46	2.95±0.86	1.23±0.50	1.06±0.68	1.46±0.91
TT	67	4.80±1.65	0.88(0.58)	1.98±0.62	2.84±1.08	1.16±0.58	1.04±0.70	1.46±1.01
*F*		0.984	−8.173	5.725	1.033	5.805	1.687	2.463
*P*		0.375	0.000	0.004	0.357	0.003	0.186	0.086

### Relative factors for serum lipid parameters

The multiple linear regression analysis showed that serum HDL-C levels and the ApoA1/ApoB ratio in both ethnic groups, TC, HDL-C, ApoB levels and the ApoA1/ApoB ratio in Han and TAG, HDL-C, LDL-C, ApoA1 levels and the ApoA1/ApoB ratio in Mulao were correlated with the genotypes (*P*<0.05–0.001; Table 5).

**Table 5:** Relationship between serum lipid parameters and relative factors in the Han and Mulao populations TC, total cholesterol; TAG, triacylglycerol; HDL-C, high-density lipoprotein cholesterol; LDL-C, low-density lipoprotein cholesterol; ApoA1, apolipoprotein A1; ApoB, apolipoprotein B; ApoA1/ApoB, the ratio of apolipoprotein A1 to apolipoprotein B.

Lipid parameter	Risk factor	*B*	Std. error	Beta	*t*	*P*
Han and Mulao						
TC	Age	0.011	0.002	0.131	5.351	0.000
	Alcohol consumption	0.079	0.040	0.047	1.976	0.048
	Diastolic BP	0.009	0.003	0.077	3.130	0.002
	BMI	0.047	0.009	0.121	5.025	0.000
	Glucose	0.052	0.016	0.075	3.155	0.002
TAG	Waist circumference	0.063	0.008	0.180	7.533	0.000
	Cigarette smoking	0.424	0.087	0.112	4.849	0.000
	Diastolic BP	0.012	0.006	0.045	1.858	0.036
	Ethnic group	−0.483	0.134	−0.083	−3.615	0.000
	Glucose	0.154	0.038	0.097	4.084	0.000
	Age	−0.004	0.005	−0.018	−0.741	0.045
HDL-C	Waist circumference	−0.009	0.002	−0.141	−4.255	0.000
	Alcohol consumption	0.085	0.019	0.119	4.397	0.000
	Gender	0.095	0.029	0.090	3.295	0.001
	Age	0.002	0.001	0.063	2. 738	0.006
	BMI	−0.013	0.005	−0.082	−2.512	0.012
	Genotype	0.088	0.017	0.117	5.049	0.000
LDL-C	Age	0.009	0.002	0.140	5.835	0.000
	BMI	0.044	0.007	0.155	6.643	0.000
	Ethnic group	0.053	0.043	0.029	1.230	0.021
	Glucose	0.026	0.012	0.051	2.112	0.035
ApoA1	Alcohol consumption	0.102	0.014	0.203	7.388	0.000
	BMI	−0.008	0.003	−0.071	−2.968	0.003
	Gender	0.025	0.020	0.034	1.238	0.021
ApoB	Waist circumference	0.012	0.001	0.198	8.584	0.000
	Ethnic group	0.152	0.022	0.158	6.882	0.000
	Glucose	0.025	0.006	0.093	4.040	0.000
ApoA1/ApoB	Waist circumference	−0.013	0.004	−0.119	−3.527	0.000
	Glucose	−0.023	0.012	−0.046	−1.899	0.048
	BMI	−0.026	0.009	−0.093	−2.792	0.005
	Alcohol consumption	0.082	0.033	0.068	2.469	0.014
	Gender	0.077	0.050	0.043	1.558	0.011
	Ethnic group	−0.062	0.042	−0.035	−1.472	0.014
	Age	−0.005	0.002	−0.086	−3.595	0.000
	Genotype	−0.013	0.030	−0.010	−0.422	0.037
Han						
TC	Waist circumference	0.017	0.005	0.110	3.159	0.002
	Age	0.006	0.003	0.068	1.906	0.037
	Alcohol consumption	0.096	0.054	0.061	1.784	0.035
	Diastolic BP	0.019	0.004	0.169	4.821	0.000
	Glucose	0.078	0.021	0.125	3.662	0.000
	Genotype	−0.134	0.058	−0.076	−2.310	0.021
TAG	Waist circumference	0.072	0.015	0.166	4.845	0.000
	Cigarette smoking	0.642	0.144	0.148	4.464	0.000
	Glucose	0.269	0.060	0.153	4.457	0.000
	Diastolic BP	0.018	0.011	0.057	1.624	0.010
	Age	−0.012	0.009	−0.050	−1.423	0.015
HDL-C	Waist circumference	−0.011	0.002	−0.158	−4.557	0.000
	Gender	0.102	0.045	0.090	2.287	0.022
	Alcohol consumption	0.068	0.029	0.093	2.385	0.017
	Genotype	0.122	0.027	0.150	4.518	0.000
LDL-C	Age	0.009	0.002	0.131	3.779	0.000
	BMI	0.044	0.009	0.158	4.734	0.000
	Glucose	0.042	0.016	0.090	2.613	0.009
ApoA1	Alcohol consumption	0.066	0.014	0.189	4.697	0.000
	BMI	−0.014	0.003	−0.170	−4.972	0.000
	Gender	−0.035	0.024	0.065	1.460	0.014
	Cigarette smoking	0.051	0.014	0.150	3.544	0.000
ApoB	Waist circumference	0.006	0.001	0.234	5.480	0.000
	Glucose	0.018	0.004	0.162	5.147	0.000
	Alcohol consumption	0.021	0.009	0.075	2.307	0.021
	Systolic BP	0.002	0.000	0.148	4.651	0.000
	BMI	0.004	0.003	0.067	1.574	0.011
	Genotype	−0.018	0.010	−0.057	−1.841	0.036
ApoA1/ApoB	Waist circumference	−0.009	0.003	−0.141	−3.134	0.002
	Systolic BP	−0.002	0.001	−0.078	−2.347	0.019
	Glucose	−0.020	0.008	−0.078	−2.419	0.016
	BMI	−0.024	0.007	−0.152	−3.475	0.001
	Cigarette smoking	0.109	0.026	0.170	4.222	0.000
	Gender	0.142	0.043	0.138	3.333	0.001
	Genotype	0.071	0.024	0.096	2.996	0.003
Mulao						
TC	Age	0.015	0.003	0.175	5.392	0.000
	BMI	0.059	0.013	0.144	4.439	0.000
TAG	Waist circumference	0.056	0.008	0.219	6.672	0.000
	Alcohol consumption	0.275	0.101	0.089	2.717	0.007
	Genotype	0.026	0.098	0.09	0.263	0.029
HDL-C	Waist circumference	−0.009	0.003	−0.147	−3.143	0.002
	Alcohol consumption	0.065	0.023	0.092	2.825	0.005
	BMI	−0.021	0.007	−0.129	−2.775	0.006
	Age	0.003	0.001	0.101	3.091	0.002
	Genotype	0.052	0.023	0.075	2.320	0.021
LDL-C	Age	0.011	0.002	0.172	5.221	0.000
	BMI	0.044	0.010	0.149	4.600	0.000
	Genotype	−0.151	0.042	−0.118	−3.572	0.000
ApoA1	Alcohol consumption	0.114	0.021	0.179	5.498	0.000
	Genotype	−0.067	0.020	−0.108	−3.295	0.001
ApoB	Waist circumference	0.014	0.002	0.191	5.847	0.000
ApoA1/ApoB	Waist circumference	−0.023	0.005	−0.166	−4.976	0.000
	Cigarette smoking	−0.064	0.057	−0.041	−1.131	0.025
	Alcohol consumption	0.115	0.060	0.070	1.931	0.045
	Genotype	−0.090	0.053	−0.056	−1.696	0.009

As shown in Table 6, when serum lipid data were analysed according to gender, serum TC, TAG, LDL-C, ApoB levels and the ApoA1/ApoB ratio in Han males; TAG, HDL-C, ApoA1 levels and the ApoA1/ApoB ratio in Han females; LDL-C and ApoA1 levels in Mulao males; and TAG, HDL-C and ApoA1 levels in Mulao females were correlated with genotypes (*P*<0.05–0.001).

**Table 6:** Relationship between serum lipid parameters and relative factors in the males and females of the Han and Mulao populations TC, total cholesterol; TAG, triacylglycerol; HDL-C, high-density lipoprotein cholesterol; LDL-C, low-density lipoprotein cholesterol; ApoA1, apolipoprotein A1; ApoB, apolipoprotein B; ApoA1/ApoB, the ratio of apolipoprotein A1 to apolipoprotein B.

Lipid parameter	Risk factor	*B*	Std. error	Beta	*t*	*P*
Han/male						
TC	Diastolic BP	0.031	0.005	0.269	5.818	0.000
	Alcohol consumption	0.014	0.064	0.010	0.217	0.028
	Glucose	0.097	0.032	0.141	3.019	0.003
	Genotype	−0.282	0.089	−0.147	−3.174	0.002
TAG	Waist circumference	0.107	0.025	0.201	4.237	0.000
	Cigarette smoking	0.681	0.227	0.139	2.992	0.003
	Diastolic BP	0.020	0.019	0.052	1.091	0.276
	Glucose	0.398	0.110	0.169	3.628	0.000
	Genotype	−0.848	0.302	−0.130	−2.811	0.005
HDL-C	BMI	−0.032	0.006	−0.265	−5.452	0.000
	Alcohol consumption	0.074	0.023	0.157	3.230	0.001
	Genotype	0.068	0.031	0.103	2.207	0.028
LDL-C	Cigarette smoking	−0.238	0.051	−0.221	−4.685	0.000
	BMI	0.033	0.012	0.125	2.645	0.008
ApoA1	Alcohol consumption	0.067	0.015	0.214	4.364	0.000
	BMI	−0.016	0.004	−0.195	−4.020	0.000
	Cigarette smoking	0.050	0.015	0.152	3.228	0.001
ApoB	BMI	0.007	0.003	0.119	2.123	0.034
	Glucose	0.024	0.005	0.199	4.493	0.000
	Alcohol consumption	0.006	0.011	0.026	0.558	0.027
	Diastolic BP	0.004	0.001	0.205	4.555	0.000
	Waist circumference	0.005	0.002	0.189	3.399	0.001
	Genotype	−0.040	0.015	−0.120	−2.753	0.006
ApoA1/ApoB	BMI	−0.047	0.007	−0.339	−7.239	0.000
	Cigarette smoking	0.091	0.026	0.158	3.461	0.001
	Alcohol consumption	0.078	0.026	0.141	2.991	0.003
	Genotype	0.084	0.034	0.109	2.433	0.015
Han/female						
TC	Alcohol consumption	−0.123	0.221	−0.026	−0.558	0.037
	Age	0.028	0.004	0.318	6.972	0.000
	BMI	0.056	0.018	0.141	3.093	0.002
TAG	Waist circumference	0.025	0.016	0.077	1.581	0.015
	Diastolic BP	0.013	0.011	0.058	1.194	0.023
	Glucose	0.134	0.053	0.121	2.548	0.011
	Genotype	−0.115	0.152	−0.036	−0.758	0.049
HDL-C	Waist circumference	−0.005	0.004	−0.053	−1.136	0.025
	Genotype	0.167	0.043	0.182	3.874	0.000
LDL-C	BMI	0.053	0.014	0.173	3.778	0.000
	Age	0.021	0.003	0.299	6.543	0.000
	Alcohol consumption	−0.179	0.172	−0.048	−1.041	0.298
ApoA1	Cigarette smoking	0.101	0.055	0.087	1.834	0.046
	Diastolic BP	−0.002	0.001	−0.100	−2.129	0.034
	Genotype	0.045	0.016	0.131	2.768	0.006
ApoB	BMI	0.016	0.003	0.221	4.966	0.000
	Glucose	0.014	0.005	0.137	2.984	0.003
	Age	0.004	0.001	0.251	5.316	0.000
	Cigarette smoking	−0.108	0.044	−0.111	−2.431	0.015
ApoA1/ApoB	BMI	−0.036	0.008	−0.206	−4.460	0.000
	Cigarette smoking	0.345	0.112	0.144	3.093	0.002
	Systolic BP	−0.002	0.001	−0.070	−1.373	0.017
	Age	−0.008	0.002	−0.197	−3.765	0.000
	Genotype	0.057	0.032	−0.080	1.754	0.008
Mulao/male						
TC	BMI	0.074	0.020	0.170	3.633	0.000
TAG	Waist circumference	0.074	0.013	0.260	5.681	0.000
HDL-C	Alcohol consumption	0.104	0.029	0.167	3.630	0.000
	Waist circumference	−0.013	0.003	−0.219	−4.759	0.000
	Age	0.004	0.002	0.100	2.180	0.030
LDL-C	BMI	0.034	0.014	0.113	2.414	0.016
	Genotype	−0.168	0.061	−0.129	−2.756	0.006
ApoA1	Alcohol consumption	0.137	0.024	0.260	5.652	0.000
	Genotype	−0.044	0.030	−0.067	−1.456	0.014
ApoB	Waist circumference	0.012	0.004	0.161	3.428	0.001
ApoA1/ApoB	Alcohol consumption	0.096	0.062	0.073	1.540	0.012
	Waist circumference	−0.016	0.006	−0.129	−2.714	0.007
Mulao/female						
TC	Age	0.018	0.004	0.204	4.437	0.000
TAG	BMI	0.040	0.025	0.074	1.577	0.015
	Genotype	−0.139	0.110	−0.059	−1.263	0.207
HDL-C	BMI	−0.034	0.007	−0.233	−5.149	0.000
	Genotype	0.080	0.028	0.128	2.831	0.005
LDL-C	BMI	0.059	0.013	0.203	4.495	0.000
	Age	0.013	0.003	0.193	4.288	0.000
ApoA1	Cigarette smoking	0.133	0.197	0.031	0.674	0.015
	Diastolic BP	−0.002	0.002	−0.047	−1.017	0.031
	Alcohol consumption	0.064	0.119	0.025	0.543	0.037
	Genotype	−0.093	0.028	−0.153	−3.297	0.001
ApoB	Glucose	0.041	0.020	0.094	2.060	0.040
	Waist circumference	0.016	0.003	0.213	4.661	0.000
	Cigarette smoking	0.606	0.257	0.107	2.358	0.019
ApoA1/ApoB	BMI	−0.017	0.024	−0.045	−0.698	0.048
	Cigarette smoking	−0.579	0.526	−0.050	−1.101	0.271
	Waist circumference	−0.024	0.010	−0.156	−2.386	0.017
	Age	−0.011	0.004	−0.133	−2.886	0.004

Serum lipid parameters were also associated with age, gender, BMI, systolic and diastolic BPs, fasting blood glucose levels, cigarette smoking and alcohol consumption in both ethnic groups (*P*<0.05–0.001, Tables 5 and 6).

## DISCUSSION

The SNP of rs10903129 near the TMEM57 locus has been associated with TC in a previous GWAS [12,13], but the association of the SNP of rs873308 near the TMEM57 locus and serum lipid levels has not been previously reported. Furthermore, the genotypic and allelic frequencies of the TMEM57 rs873308 SNP have not been reported previously in different racial/ethnic and sex groups. In the present study, we revealed that the genotypic and allelic frequencies of TMEM57 rs873308 SNP were not different between the Chinese Han and Mulao populations, but the allelic frequencies were different between males and females in the both ethnic groups (*P*<0.05), the frequency of the T allele was higher in females than in males. There was no difference in the genotypic frequencies between males and females in both ethnic groups. These results suggest that the prevalence of T allele frequency of TMEM57 rs873308 SNP may have sex-specificity in our study populations.

In the present study, our findings also suggest that there may be a racial/ethnic- and sex-specific association of the TMEM57 rs873308 SNP and serum lipid levels. The T allele carriers had lower TC and ApoB levels and higher HDL-C levels and the ApoA1/ApoB ratio in Han; lower TAG, LDL-C, ApoA1 levels and the ApoA1/ApoB ratio and higher HDL-C levels in Mulao; lower TC, TAG and ApoB levels and higher HDL-C levels and the ApoA1/ApoB ratio in Han males; lower TAG levels and higher HDL-C, ApoA1 levels and the ApoA1/ApoB ratio in Han females; lower LDL-C and ApoA1 levels in Mulao males; and lower TAG and ApoA1 levels and higher HDL-C levels in Mulao females than the T allele non-carriers. However, these differences may also be related to the variations in examined populations, including healthy, hypercholesterolemic and overweight/obese subjects; modulating environmental factors such as diet or under medication. Therefore, this association of the TMEM57 rs873308 SNP and serum lipid levels needs to be further studied with larger sample size.

Sex has important influences on neural function and disease, as well as responses to metabolic stressors. Sex differences in dyslipidaemia have been noted for many years. It is commonly accepted that androgens induce changes in lipid concentrations that would predispose towards CHD, whereas oestrogens are held to have opposite effects [25]. Oestrogens share structural similarities with vitamin E and other lipophilic antioxidants and are thus able to function as scavengers for lipid peroxyl radicals and interrupt the chain reaction of lipid peroxidation. Oestradiol at physiological levels has an antioxidant capacity that is independent of its effects on blood lipid concentrations, an action that may be of anti-atherogenic importance [26]. The oxidative modification of LDL (low-density lipoprotein) is important in the pathogenesis of atherosclerosis, and oestrogen has been shown to inhibit copper and cell-mediated oxidation of LDL *in vitro* [27]. Several studies in postmenopausal women who have been treated with oral and transdermal oestrogens have shown decreased susceptibility of LDL particles to oxidation that is independent of the lipid-lowering effects. Oestrogens also protect HDL from oxidation, an effect that should preserve the beneficial functions of HDL, including the protection of LDL from oxidation. Although the effects of gonadal hormones on neural function and lipid metabolism that regulate serum lipid levels are considered contributing factors, the reasons for sex differences in dyslipidaemia are still not well understood. Other unknown genetic factors may also be involved in determining this complex status. In the present study, we compared the genotypic and allelic frequencies between males and females in both ethnic groups. The results showed that the frequency of T allele was lower in males than in females in both ethnic groups. These findings suggest that the genetic variation of T allele of TMEM57 rs873308 SNP is lower in men than in women in our study populations. The difference in serum lipid levels between males and females in the both ethnic groups might partly result from different TMEM57 rs873308 SNP.

In addition to the effects of the TMEM57 rs873308 SNP on serum lipid levels, we also showed that several environmental factors such as age, gender, BMI, waist circumference, systolic and diastolic BPs, blood glucose, alcohol consumption and cigarette smoking were associated with serum lipid levels in both ethnic groups. Although rice and corn are the staple foods in the both ethnic groups, Mulao people live in an isolated environment and share local similar recipes. They consume too many acidic and spicy dishes, local bean soy sauce, pickled vegetables and animal offals which contain abundant saturated fatty acid. Many studies stated that diet alone can account for the variability on serum lipid levels [28–30]. It has been reported that diet rich in PUFAs (polyunsaturated fatty acids), monounsaturated fatty acids, carbohydrates and even saturated fatty acid, stearic acid can reduce LDL-C levels [31,32]. As our experimental results showed different dietary habits, lifestyles and environmental factors probably further modify the effect of genetic variation on serum lipid levels in our study populations. Many studies also stated that daily eating habits can strongly influence the serum levels of ApoB, ApoA1 and their ratio, and which in turn can result in the risk of CHD [33–35]. The current study might be partly attributed to the difference in daily eating habits between the Mulao and Han populations.

There are still several limitations in this study. First, the general characteristics of the both ethnic groups are different. Although sex, age, BMI, BP, alcohol consumption and cigarette smoking have been adjusted for the statistical analysis, we could not completely eliminate the effects of these factors on serum lipid levels among different genotypes in both ethnic groups. Second, the diet was not adjusted for the statistical analysis. In the present study, however, the diet in this isolated population is consistent throughout the year and among individuals because of the Mulao's reliance on a limited number of locally available food items. Third, it is well known that both oestrogen and menopause can influence serum lipid levels. In the present study, however, we did not compare the difference in serum lipid levels between the premenopausal and postmenopausal women in the both ethnic groups because of the relatively small samples. Finally, it is clearly established that serum lipid levels are regulated by multiple environmental and genetic factors, and their interactions. Although we have detected the association of the TMEM57 rs873308 SNP and several environmental factors with serum lipid profiles in this study, there are still many unmeasured environmental and genetic factors and their interactions. Thus, the interactions of gene–gene, gene–environment, and environment–environment on serum lipid levels remain to be determined.

### Conclusions

The present study showed that the TMEM57 rs873308 SNP and several environmental factors were associated with some serum lipid parameters in the Chinese Han and Mulao populations, but the associated trends of the SNP and serum lipid parameters are different. There is a sex-specific association of the TMEM57 rs873308 SNP and serum lipid parameters in both ethnic groups.
